# Hygrothermal Aging Characteristics of Silicone-Modified Aging-Resistant Epoxy Resin Insulating Material

**DOI:** 10.3390/polym13132145

**Published:** 2021-06-29

**Authors:** Yongqiang Wang, Zhuo Zeng, Meng Gao, Ziye Huang

**Affiliations:** Hebei Provincial Key Laboratory of Power Transmission Equipment Security, School of Electrical Engineering, North China Electric Power University, Baoding 071003, China; ncepugideon@163.com (Z.Z.); 13734374729@126.com (M.G.); hzy1053652698@163.com (Z.H.)

**Keywords:** silicone-modified epoxy resin, dielectric properties, hygrothermal aging

## Abstract

To study the improvement effect of silicone materials on the hygrothermal resistance of epoxy resin and the aging mechanism of silicone-modified insulation materials under hygrothermal conditions, diphenylsilanediol was added to epoxy resin as a modifier in various quantities to synthesize silicone-modified epoxy resin, and a hygrothermal aging test was carried out. Water sorption, surface contact angles and dielectric properties of the insulation material were measured, and scanning electron microscope (SEM), Fourier-transform infrared spectrometry (FT-IR) and frequency domain spectroscopy (FDS) were used to analyze the results. The results showed that under 10 wt%, the silicone-modified insulation materials exhibited lower absorption rate and better dielectric properties, including lower dissipation factors and lower dielectric constant during the hygrothermal aging process, while epoxy resin modified with excessive silicone material tend to show worse dielectric performance. Closer analysis found that diphenylsilanediol decreases the size of the cracks within the material during hygrothermal aging, indicating that cracks generated during the hygrothermal aging process may be the reason for the worse dielectric performance, and diphenylsilanediol improves the hygrothermal aging resistance mainly by slowing down the generation and growth rate of cracks. FT-IR results confirmed the existence of hydrolysis and found that the rate of hydrolysis does not change with the content of diphenylsilanediol. FDS results also indicated that modified materials contain less dipoles after hygrothermal aging.

## 1. Introduction

Epoxy resin is widely used in electrical equipment due to its high electrical strength, excellent chemically stability, solvent resistance and easy processing. With the continuous development of the power industry, the capacity of electrical equipment is increasing, which puts forward higher requirements for the performance of insulating materials. However, under high operating temperature, especially in high humidity areas, such as coastal areas, water could erode epoxy resin insulation materials, affecting the dielectric properties and mechanical properties of insulating materials [1,2]. According to statistics, the main reason for power equipment failures is electrical. The deterioration of dielectric properties could lead to insulation failure and tracking, which seriously threaten the safe and stable operation of electrical equipment. Therefore, it is necessary to improve the hygrothermal aging resistance of insulating materials and study their hygrothermal aging characteristics.

Plenty of researchers have focused on the effect of hygrothermal conditions on polymers [3,4,5]; it has been reported that elevated temperatures could accelerate the diffusion of water molecules into the material, and the bounded water could lead to hydrolysis and increase in pore content. Hydrolysis and pores may pose a serious threat to the durability of epoxy resin materials. Experiments conducted by researchers have reported an obvious decrease in the mechanical properties of epoxy resin after hygrothermal aging.

Though the detrimental effect of hygrothermal aging has been acknowledged, the mechanism of hygrothermal aging is not completely understood. There are two different views in terms of the hygrothermal aging mechanism of epoxy resin materials. The first one believes that water molecules exist in epoxy resin in two forms: free water and bound water. Free water usually aggregates in the voids and cavities of the sample, while bound water interacts with the polar groups in the epoxy resin, resulting in a tight connection. Water disrupts the hydrogen-bonding network, thus reducing the performance of epoxy resin materials [6,7,8]; the other view is that the water disperses in resin homogeneously and that there is no such thing as free water or bound water in epoxy resin. The mobility of water molecules is between free water and bound water, and water has no significant impact on the hydrogen-bonding network in epoxy resin; it only acts as a plasticizer in the resin system [9,10].

Owing to the stability and flexibility of the Si–O bond, silicone compounds show advantages, such as good high-temperature resistance, good toughness and hydrophobicity. These properties can compensate for the shortcomings of epoxy resin materials. Silicone materials also have good electrical properties, which make them suitable for the modification of insulating materials. Previous work has found that silicone material could increase the stability of epoxy. Chen et al. [11] used (γ-aminopropyl)methyldimethoxysilane to synthesize modified epoxy resin. The results indicated significant improvements in the corrosion resistance of acid, alkali and salt after modification. Wen et al. [12] introduced a silicon element and functional groups into cycloaliphatic epoxy resin and increased the compatibility between the matrix and modifier using phenylmethyl silicone resin. It was found that modified materials showed better thermal stability, UV resistance and lower water sorption.

Apart from the work mentioned above, researchers also found silicone material promising in improving materials’ performance under hygrothermal conditions specifically. Song [13] conducted a hygrothermal test on epoxy resin composite materials modified with different contents of polyhedral oligomeric silsesquioxane (POSS). It was found that the water sorption of epoxy resin composite materials followed a Fickian diffusion model, and the sorption of the epoxy resin composite increased as the POSS contents increased; the modifier helped to improve the retention rate of the interlaminar shear strength of the materials after hygrothermal aging. Badu et al. [14] studied the effect of a hygrothermal environment on properties of a silane coupling agent-modified resin and suggested that the covalent bond produced by silane hydrolysis slows down the degradation of resin at low silane content, while higher wt% of silane may accelerate the degradation process. Wei and Wang [15] modified phenolic resin insulation material with silane coupling agent KH-550. Their experiments showed that grafting KH-550 onto phenolic resin can effectively reduce water absorption and increase bulk resistance of phenolic resin. After hygrothermal aging, silicone-modified phenolic resin still exhibits a higher bulk resistivity and bond strength than pure resin.

Most research related to the hygrothermal degradation of epoxy resin focused on its mechanical properties. Yet, a limited amount of research has focused on the changes of electrical performance, which is more crucial for insulating materials, of silicone-modified resin during hygrothermal aging process. Furthermore, previous work by other researchers managed to explain how hygrothermal degradation undermines epoxy resin’s performance, but the mechanism about how silicone materials improve the anti-aging performance of epoxy resin is still unclear. To investigate the dielectric properties of silicone-modified epoxy resin insulating materials during the hygrothermal aging process and gain more insight into the mechanism, in this paper, bisphenol A epoxy resins modified with different amounts of diphenylsilanediol were synthesized, and their water sorption, changes of partial discharge initiation voltage (PDIV) and dielectric properties were studied. Meanwhile, the micromorphology of the outer surface and cross-section was observed, and the changes of hydrophobicity of the materials with aging time were measured. Based on the above experimental data, the performance degradation mechanism of silicone-modified epoxy resin was discussed.

## 2. Materials and Methods

### 2.1. Materials and Sample Preparation

The epoxy resin used in this experiment was E-51 epoxy resin manufactured by Shanghai Resin Factory (Shanghai, China). The curing agent was methyl hexahydrophthalic anhydride provided by Guangzhou Zhonggao New materials Co., Ltd. (Guangzhou, China); the accelerator was DMP-30. The silicone modifier was diphenylsilanediol produced by Hongcheng Bio Company (Wuhan, China) with a purity of 95%; the catalyst was dibutyltin dilaurate.

**Figure 1:**
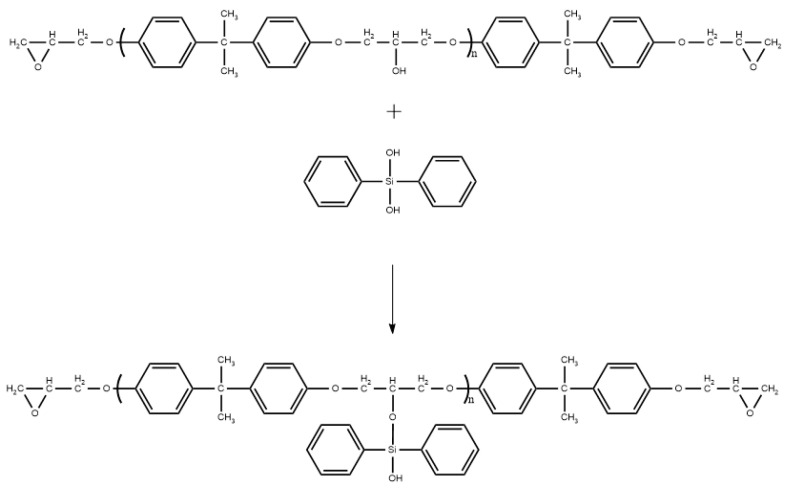
Grafting reaction.

**Figure 2:**
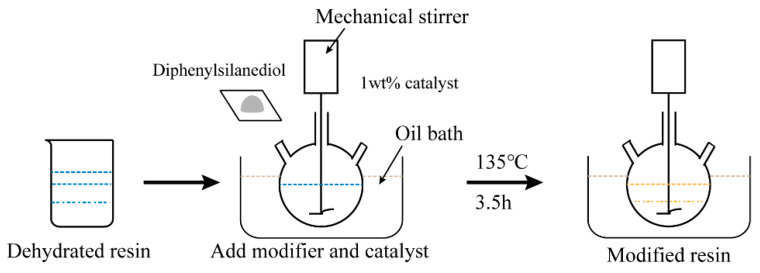
Scheme of the modification.

In this paper, epoxy resin was first put under vacuum at 80 °C for 2 h to fully remove the moisture absorbed in the resin; then, the epoxy resin was added into the three-port reactor, and a certain amount of diphenylsilanediol and 1 wt% of dibutyltin dilaurate were added as a catalyst. The contents of the diphenylsilanediol used in this experiment were 0 wt%, 5 wt%, 10 wt% and 15 wt%, respectively, and the samples were denoted as m0, m5, m10 and m15; the three-port reactor was placed in an oil bath, heated to 135 °C and stirred continuously for 3.5 h to complete the grafting reaction. The grafting reaction and its scheme are shown in Figure 1 and Figure 2.

After grafting, solid diphenylsilanediol disappeared, and the epoxy resin turned from colorless to yellowish. The epoxy equivalent weight (EEW) of the reactants was determined according to the GB-T1677-2008 standard, and the results are surmised in Table 1 below. The EEW of the epoxy resin after modification barely changed, indicating that the epoxy group of the epoxy resin was not consumed during the modification, thus confirming the reaction shown in Figure 1. The results in Table 1 also show that there is no need to change the ratio of the curing agent for the four groups of samples; therefore, the mass ratio of the epoxy resin, curing agent and accelerator for all four groups of samples was set to be 100:80:0.5.

The fully mixed sample was poured into a square silicone rubber mold with a length of 2 cm and a depth of 8 mm. The mixture was put under high vacuum at 60 °C for 1 h to fully degas. The epoxy resin was cured under 100 °C for 2 h, and then under 130 °C for 2 h. Finally, samples were heated to 150 °C for 1 h for post-curing. For samples prepared for partial discharge tests, needle electrodes with a diameter of 0.3 mm were embedded in the sample, and the distance between the needle electrode and the bottom of the epoxy resin sample was 5 mm.

Before aging, the samples were placed in an oven at 120 °C for 48 h to fully remove moisture, by-products and internal residual stress. The samples were immersed in water at 90 °C during hygrothermal aging. The whole hygrothermal aging lasted for 32 days.

### 2.2. Water Sorption

Percent weight change or water sorption was calculated from

(1) $$m\%=\frac{m_{1}-m_{0}}{m_{0}}\times 100\%$$

where $m_{0}$ is the dry initial weight measured instantly after the samples were taken out of the oven, and $m_{1}$ is the weight of the specimen measured during hygrothermal aging.

The samples used in the water sorption test were 25 mm × 25 mm × 4 mm cubes. Samples were aged according to the condition described in Section 2.1. Weighting was carried out periodically, water on the samples’ surface was wiped with a clean cloth before weighting. For each group of samples, five samples were prepared, and the mean mass changes were calculated as water sorption.

### 2.3. SEM Analysis

This paper used EM–30 plus scanning electron microscope (SEM) (COXEM, Daejeon, Korea) to observe the outer surface and cross-section of the samples at different stages. Before outer surface observation, the surface of the epoxy resin block was wiped by a dust-free cloth dipped in diluted hydrochloric acid to minimize the influence of sediments from water. After that, the sample was rinsed with water and wiped dry. The cross-section was obtained by breaking the epoxy resin block, and it was observed without further treatment.

### 2.4. Partial Discharge Initiation Voltage (PDIV)

**Figure 3:**
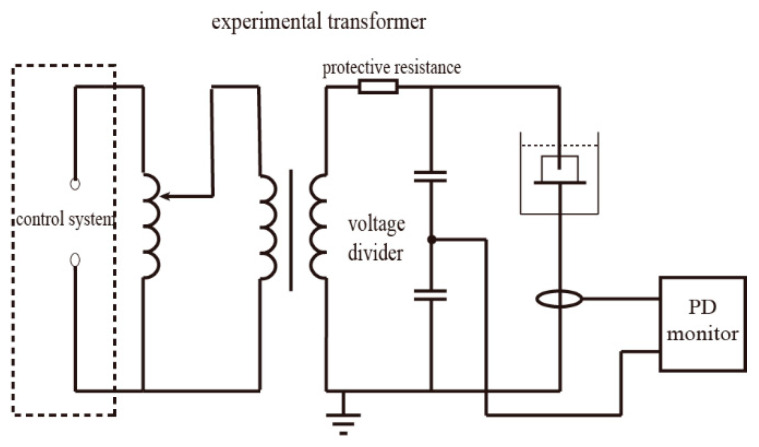
Experimental platform for partial discharge.

The experiment platform for the partial discharge test is shown in Figure 3.

The partial discharge test platform consists of an AC high voltage control system and a partial discharge detection system. Protective resistance used on the platform is 10 kΩ, and the capacitance of the capacitive divider is 1000 pF. The partial discharge detection system used an HCPD-2622 digital partial discharge (PD) detector to detect partial discharge signals. The detection system has a sampling frequency of 20 MHz.

The samples aged under hygrothermal conditions for 0/4/8/16/32 days were taken, and water on the surface was dried with absorbent paper. During the experiment, the voltage was increased at a rate of 1 kV/min, and the voltage was maintained after the discharge signal appeared. If the discharge signal remained stable for 10 min, this voltage was recorded as the initial discharge voltage U.

### 2.5. Contact Angle

A JC2000DM contact angle measuring instrument (POWEREACH Inc., Daejeon, Korea) was used to measure the surface contact angles of the four groups of epoxy resin samples after they were hydrothermally aged for 0/4/8/16/32 days. The experimental operation standard was according to GB/T 30693–2014. The experimental operation referred to GB/T 30693–2014.

### 2.6. FT-IR

Four groups of cured epoxy resin before and after aging for 32 days were pulverized with an electrical grinder. The powder was mixed with potassium bromide with mortar and was pressed into thin disks. The mass ratio between the potassium bromide and epoxy resin was about 16:1. Bruker TENSOR II (Bruker, Karlsruhe, Germany) was used to analyze the samples, and the wavenumber range was between 500 cm^−1^ and 2000 cm^−1^.

### 2.7. FDS

The frequency domain dielectric spectra were tested with an IDAX-300 Insulation Diagnostic Analyzer from Megger, Sweden. The non-grounded test mode with high accuracy was used, and the test voltage was set at 141 V. The applied electric field frequency range was 1 mHz–1 kHz. During the test, the sample was placed between two plate electrodes.

## 3. Results and Discussion

### 3.1. Water Sorption

**Figure 4:**
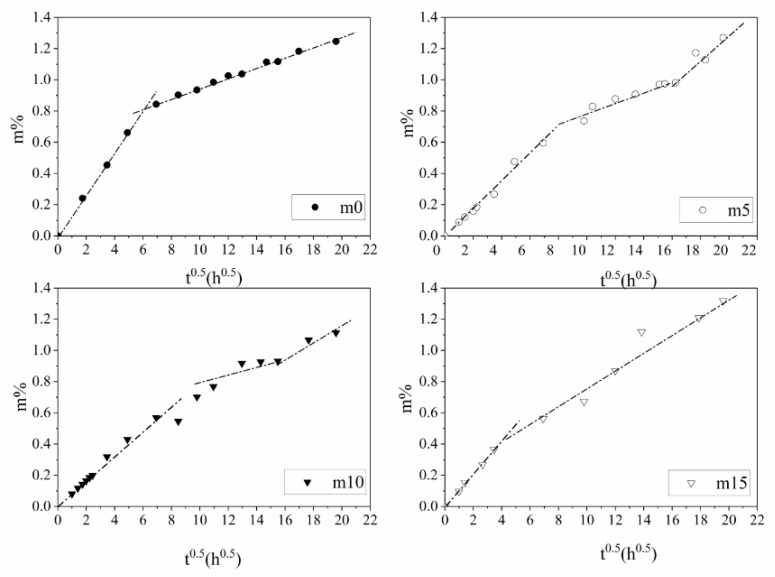
Results of the water sorption of four groups of samples with different mass fractions of silicone modifier.

The results of the water sorption of the four groups of samples with different mass fractions of the silicone modifier (m0, m5, m10 and m15) are shown in Figure 4.

The water sorption curves shown in Figure 4 can be divided into two categories: The first category (hereinafter called Case I) includes unmodified m0 and m15. The water absorption process of Case I can be divided into two stages—fast water absorption stage and slow water absorption stage. Both stages showed a linear relationship between water sorption and $\sqrt{t}$, but the slope of the first stage is greater than that of the second one. The second category (hereinafter called Case II) includes m5 and m10, whose water absorption curve can be divided into three stages. The first and the third stage of Case II are similar to the first and second stage of Case I, which can also be called the fast water absorption stage and the slow water absorption stage. The noticeable difference between Case I and Case II is that there is a pseudo-saturation stage between the first stage and the second stage, where the slope is much smaller than that of the fast and slow water absorption stage.

As for the diffusion process of water in the epoxy resin system, the most simple and widely used mathematical model is the Fickian diffusion model [16]. The Fickian diffusion model assumes that the water in the resin system exists in the form of free water. Water molecules diffuse through the long-chain molecules of resin under the concentration gradients’ force, and the system finally reaches equilibrium. The typical Fickian absorption-time square root curve consists of two stages. The Fickian diffusion model suggests the samples first experience a fast water absorption stage where the water uptake grows linearly with the square root of time, and they finally reach the saturation stage where the water uptake of the samples remains stable.

The Fickian diffusion model can be described by the following expression [17]:

(2) $$\frac{m}{m_{\infty }}=1-\frac{8}{\pi^{2}}\overset{\infty }{\underset{n=0}{\sum }}\frac{1}{{\left(2n+1\right)}^{2}}\exp\left(-\frac{\pi^{2}{\left(2n+1\right)}^{2}}{e^{2}}\right)Dt$$

where $m_{\infty }$ is the water absorption rate of epoxy resin in the saturation state; *D* is the coefficient of water diffusion, which is proportional to the slope S of the water sorption curve at the origin. The relation between *D* and *S* can be expressed by the following expression:

(3) $$D=\frac{S^{2}\pi }{16}$$

**Figure 5:**
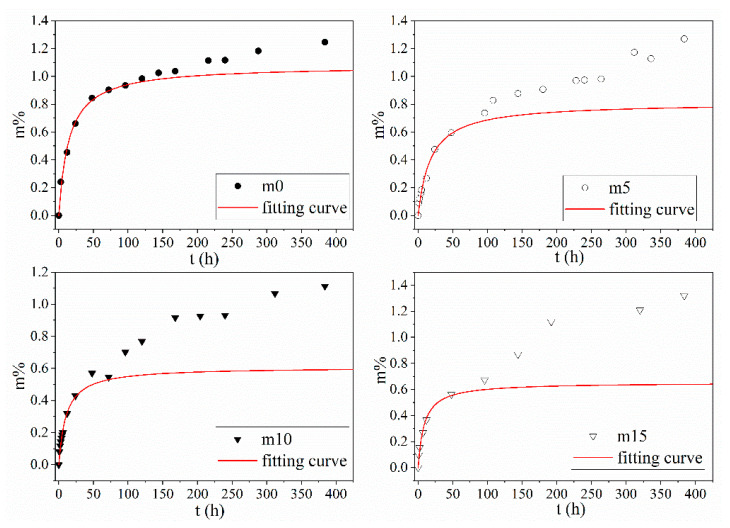
Fickian fitting results for the four groups of samples.

In the experiment, none of the four groups of the samples reached the final saturation stage, but they all followed the Fickian diffusion law during the fast water absorption stage; therefore, the Fickian model can be used to analyze the free diffusion of water in the epoxy resin system. The slope S at the origin of the four groups of resins and the fitting result for water absorption in a saturated state are shown in Table 2; the fitting results are shown in Figure 5.

As shown in Table 2, under 10 wt%, the silicone material slowed down the diffusion process, and the water absorption rate in the saturated state decreased. It is generally accepted that diffusion of water molecules and the water absorption rate in the saturated state in the Fickian model are related to the molecular polarity, free volume, hydrogen bond and other properties of the material [18]. From the perspective of molecular polarity, the encapsulation of alcohol hydroxyl groups during the synthesis and the symmetrical structure of diphenylsilanediol reduces the polarity of the insulation material. From the perspective of free volume, after the grafting reaction, the side chain of the epoxy resin becomes more flexible; the movement of the side chain may prevent the combination of water and nanopores and hinder the diffusion and storage of water molecules in the resin system. From the perspective of the hydrogen bond, the grafting reaction consumes hydroxyl groups; therefore, the binding ability of epoxy resin molecules to water molecules decreases, and, thus, the saturated water absorption of the epoxy resin system further decreases. The increase in diffusion rate and water absorption rate in the saturated state at 15 wt% may have two possible explanations: (1) The incomplete grafting reaction. Unreacted diphenylsilanediol molecules may become the impurities within the system, thus reducing the performance of the insulating material. (2) The polymerization between diphenylsilanediol molecules. Long side chains may make the structure less compact and increase the free volume within the material.

The water absorption curve in the experiment continued to rise after the fast water absorption period and significantly exceeded the saturated water absorption predicted by the Fickian model. The reasons are the following: (1) In addition to free water, there is bound water in the epoxy resin system, which is not considered by the Fickian model. (2) In addition to the Fickian diffusion, the water molecules can also enter epoxy resin by rapid transmission through macropores and the slow absorption process through micropores. In m0 and m15, some microcracks were generated on the surface, and water entered the samples through the microcracks quickly. Therefore, the moisture content of the four samples reached a higher value than that predicted by the Fickian model at the end of the fast water absorption period. After the rapid water absorption period, the cracks of m0 and m15 continued to grow and become the dominant factors for subsequent water absorption. In m5 and m10 samples, the cracks were still not obvious in the pseudo-saturation stage. At this time, the moisture content in the pores of m5 and m10 approached saturation; thus, the water absorption rate of the samples decreased rapidly, forming a platform called the pseudo-saturation period. When m5 and m10 reached 16 h^0.5^, the cracks in the sample began to occur. The samples then reached the third stage of the rate-controlling stage, and the diffusion of water into the macropores appeared again; therefore, the water absorption started to increase again but at a slower rate compared to the fast absorption period.

### 3.2. Partial Discharge Inception Voltage

**Figure 6:**
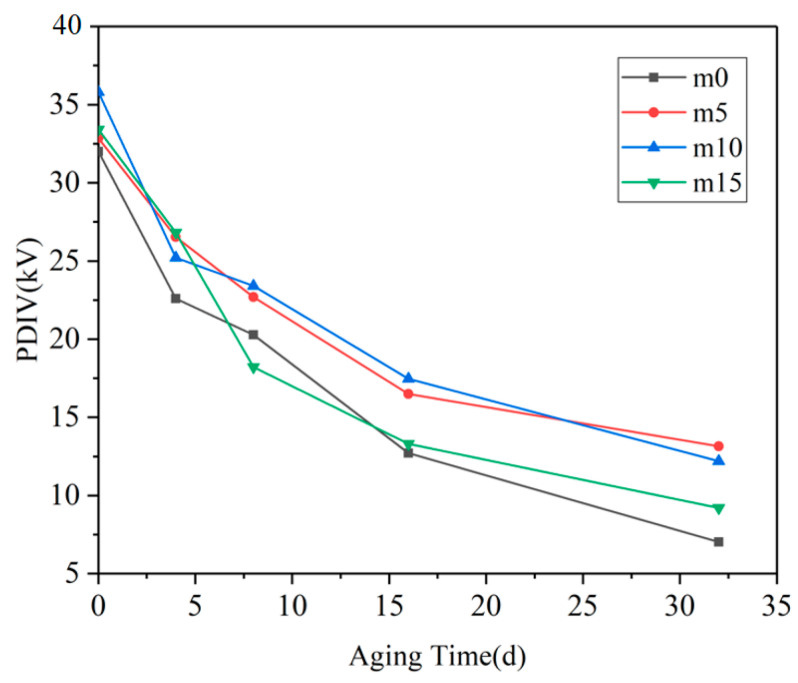
The variation of PDIV with the aging time.

The partial discharge inception voltage results of the four groups of samples are shown in Figure 6 below.

When the samples were not aged, the PDIV data of the four groups were quite close. With the progress of hygrothermal aging, the PDIV of the four groups of samples decreased significantly, and the fastest decline was in the range of 0 days–4 days, and the slowest decline was after 16 days. The PDIV of m0 and m15 (Case I) from 8 days to the end of the experiment was significantly lower than that of m5 and m10 (Case II) at the same aging time. It can be observed that m5 and m10 showed similarity in PDIV; the possible explanation for this phenomenon may be that the optimal filling content of diphenylsilanediol may be between 5 wt% and 10 wt%. At 10 wt%, this filling content may have passed the optimal filling content; therefore, the PDIV of m10 may decline to a level that is similar to that of m5.

The PDIV of insulating materials is mainly determined by the nature of the material itself and internal defects. At the early stage of hygrothermal aging, the rapid decline of the PDIV of epoxy resin material is mainly due to the change of the dielectric constant of the material. One of the reasons for the partial discharge phenomenon in insulating materials is distortion of the electric field at the inevitable tiny pores generated during production. The dielectric constant of water is very high ($\varepsilon_{r}=80$); therefore, the dielectric constant of epoxy resin increases as water diffuses into the resin system [19]. The gap between the dielectric constant of the material and the tiny pores further expands, resulting in the aggravation of the local electric field distortion at the pores and the decrease in PDIV. At day 4, the PDIV value of m0 was the lowest because its coefficient of water diffusion was higher than that of the other three groups, indicating that m0 absorbed the greatest amount of water.

After 8 days of hydrothermal aging, although water could still be absorbed into the material through hydrolysis and diffusion and the dielectric properties of the material continued to degrade, the number and size of the pores in the material gradually became the main factors. At day 16, the PDIV of the four groups of materials was less than half of that before aging, indicating that there were more pores and defects inside. The above analysis shows that after 16 days of hygrothermal aging, the main changes that happened in the material were the growth and coalescence of cracks and pores. The reason for the slowing down of the PDIV curve after 16 days may be that there are many pores that are large enough to cause PD, and the further expansion of these pore sizes has a smaller effect on the PDIV of the material.

### 3.3. SEM Analysis

According to the above analysis, in this experiment, the hygrothermal degradation process of Case I and Case II was slightly different. Therefore, in this part, the SEM images of samples of m0 and m10 were selected to represent Case I and Case II, respectively, and show the damage of hygrothermal aging on the two types of epoxy resin systems.

**Figure 7:**
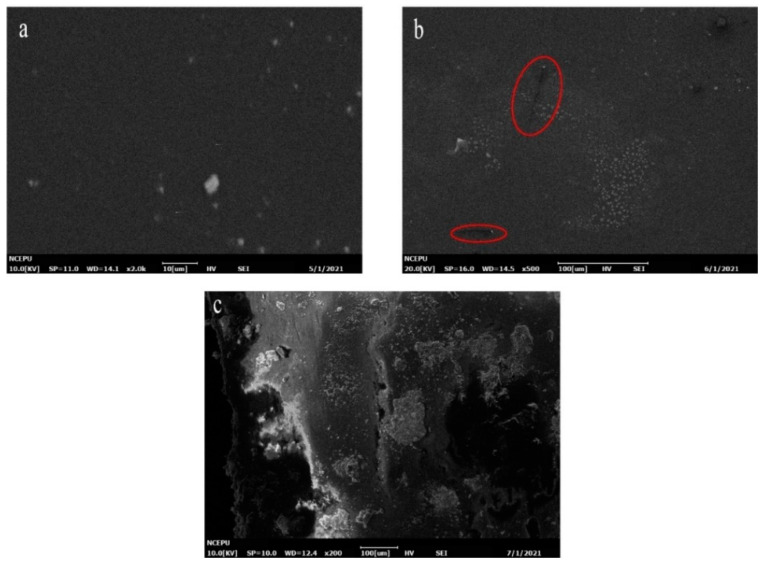
Micromorphology of the outer surface of m0. (**a**) Unaged; (**b**) aged for 4 days; (**c**) aged for 16 days.

**Figure 8:**
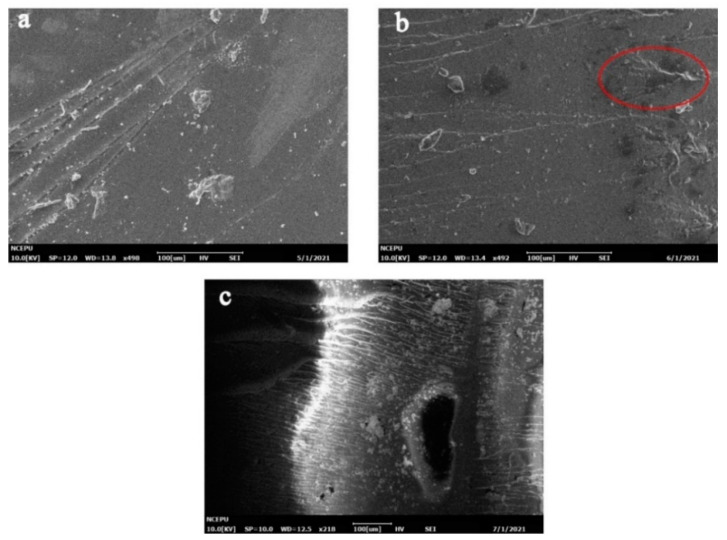
Micromorphology of the cross-section of m0. (**a**) Unaged; (**b**) aged for 4days; (**c**) aged for 16 days.

For Case I samples, the SEM images of 0/4/16 days of aging were selected to represent the three key time points of the epoxy resin material: unaged, at the end of the rapid water absorption period and the slow water absorption period. Figure 7 and Figure 8 show the micromorphology of the outer surface and the cross-section of group m0, respectively, after 0/4/16 days of hygrothermal aging.

As shown in Figure 7, before aging, the outer surface of the resin material was smooth, and no obvious defects were found. When the fast water absorption stage ended (4 days), small cracks and clustered white spots could be observed, as shown in Figure 7b. When the hygrothermal aging developed to 16 days, the damage of water on the resin surface was more obvious, and large-scale cracks and holes could be observed on the material surface.

In Figure 8, it can be observed that with the progress of aging time, inhomogeneous black blotches began to appear on the resin section. At day 16, large-scale holes could be observed inside the resin system.

**Figure 9:**
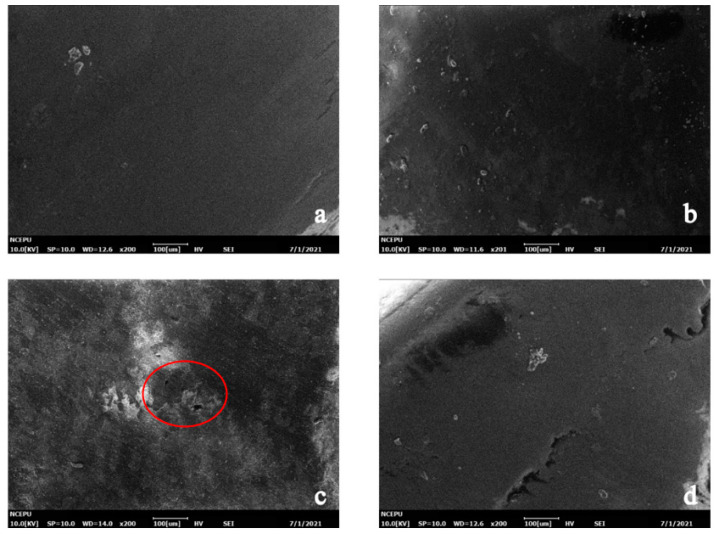
Micromorphology of the outer surface of m10. (**a**) Unaged; (**b**) aged for 4 days; (**c**) aged for 16 days; (**d**) aged for 32 days.

For Case II samples, the SEM images of 0/4/16/32 days of aging were selected to represent the four time points (unaged, end of the fast water absorption stage, end of pseudo-saturation stage and during the slow water absorption stage, respectively). The SEM images of the outer surface of m10 silicone-modified epoxy resin after 0/4/16/32 days hygrothermal aging is shown in Figure 9. There were no obvious cracks on the resin surface at day 0 and day 4. At the end of the pseudo-saturation stage (day 16), obvious holes were observed on the resin surface. In the third stage (day 32), longer and deeper cracks due to water erosion could be observed.

**Figure 10:**
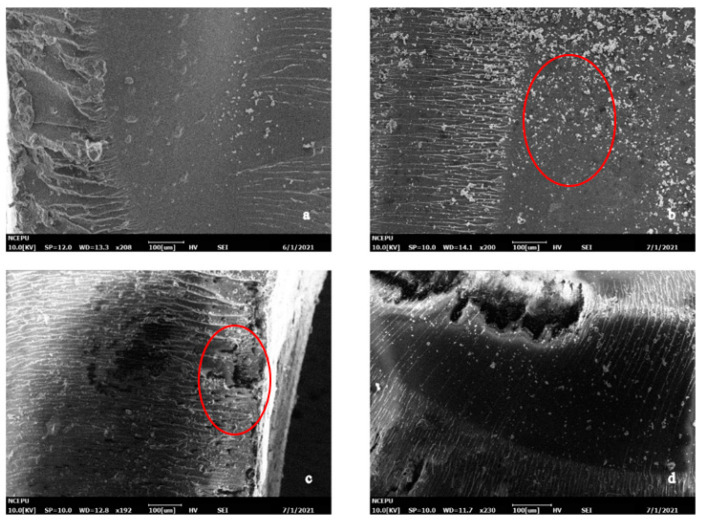
Micromorphology of the m10 cross-section. (**a**) Unaged; (**b**) aged for 4 days; (**c**) aged for 16 days; (**d**) aged for 32 days.

Figure 10 shows the microstructure of the cross-section of the m0 sample after 0/4/16/32 d of hygrothermal aging. From the surface morphology of the cross-section, no obvious phase separation phenomenon was observed in the SEM images, indicating that the compatibility between the modified materials and epoxy resin was good. At day 0 and day 4, the SEM images of the m0 and m10 samples were similar. Similarly to Figure 8b, black blotches also appeared in Figure 10b at the end of the fast water absorption period. When the hygrothermal aging reached day 16, there were also obvious pores in Figure 10c, but the size was relatively small compared with the pores in Figure 8c. At the same time, it can be found from Figure 10c that the pores were more likely to distribute at the interface between the cross-section and the outer surface, and there are signs of small holes merging into large holes, which proves that cracks develop and expand from the surface to the inside. By comparing the pore size in the cross-section of Figure 10c,d, it can be observed that the pore size in Figure 10d is significantly larger than that in Figure 10c, which proves that the growth and merger of internal pores continued in the third stage of hygrothermal aging of Case II resin.

Comparing Figure 7 and Figure 9, it can be observed that at the same time point, the damage on the outer surface of the m0 resin sample was more serious than that of the m10 resin sample, and the generation and development speed of cracks and pores on the surface of the m0 epoxy resin sample was significantly higher than that of m10. There were already small cracks at the end of the fast water absorption period in m5, while obvious changes only appeared at the surface of m10 at the end of the pseudo-saturation period. In Figure 7c, the pore diameter of the outer surface of the m0 sample was about 80 μm, while in Figure 9c, the pore diameter of the outer surface of the m10 sample was only about 20 μm and only developed to about 80 μm at day 32.

Comparing Figure 8 and Figure 10, the micromorphology changes on sections of m0 and m10 were similar to the changes that happened on the external surface, and the deterioration rate of m0 was higher than that of m10. The inhomogeneous black blotches observed in Figure 8b and Figure 10b may be due to the inhomogeneous cross-linking density inside the epoxy resin material resulting in different diffusion rates of water in different regions of the epoxy resin [20].

**Figure 11:**
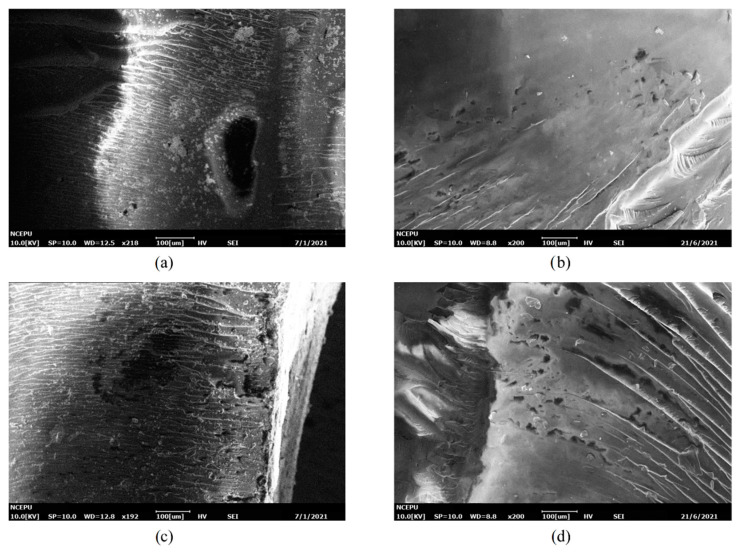
Micromorphology of the cross-section of samples aged for 16 days. (**a**) m0; (**b**) m5; (**c**) m10; (**d**) m15.

Comparisons between Figure 7, Figure 8, Figure 9 and Figure 10 reveal that silicone material slows down the generation and development rate of cracks and pores within the material. To better present the aging mechanism, the micromorphology of the cross-section at different filling contents of diphenylsilanediol at day 16 of aging is given in Figure 11.

From Figure 11, it can be observed that at day 16, the sizes of the cracks of m5, m10 and m15 are significantly smaller than that of m0. The size of the cracks of m5 is similar to that of m10, about 20 μm–30 μm, while in Figure 11d, holes tend to merge into long cracks. This difference can be explained from chemical and physical perspectives. From the chemical perspective, the damage made by water is mainly through hydrolysis, which mainly occurs near the surface of the resin material. Hydrolysis can break the resin macromolecule chain and destroy the resin matrix. The chemical bonds in the diphenylsilanediol are more stable than those in the epoxy resin, making it more difficult to hydrolyze. From the physical point of view, the cracks may grow when free water molecules inside the pores change into vapor, causing a rise of internal stress inside the pores. It has been reported elsewhere that the addition of silicone materials can improve the mechanical properties of materials [21], and the addition of diphenylsilanediol may improve the ability of the material to withstand internal stress by transferring internal stress and increasing the molecules’ flexibility [22,23]; this means that more energy may be needed to cause stress concentration that is severe enough to break the material, thus inhibiting the generation and development of cracks and pores. These observations prove the assumption in Section 3.2 that the size of cracks and holes may play an important role in the changes of PDIV.

### 3.4. Analysis of Contact Angle

**Figure 12:**
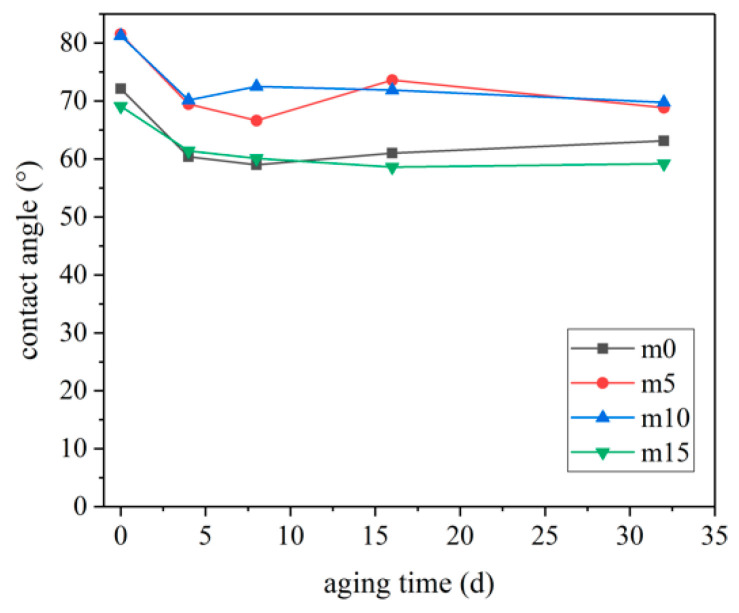
The change of contact angle of materials with hygrothermal aging.

**Figure 13:**
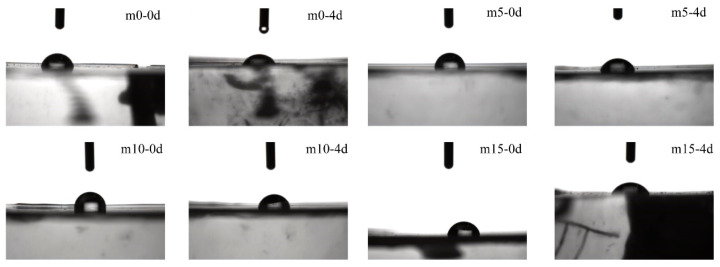
Photos of contact angle measurement.

The surface contact angles of the four groups of samples with different aging times are shown in Figure 12, and the photos of the samples are shown in Figure 13.

When unaged, the contact angles of m0 and m10 samples were close, about 10° higher than those of the m0 and m15 samples. During 0 day–4 days of hydrothermal aging, the surface contact angles of the four groups of epoxy resin samples decreased significantly, with an average decrease of about 12°. It should be noted that the contact angles of m5 and m10 after 4 days of hygrothermal aging were still close to that of m0 and m15 before aging. After 4 days, the surface contact angles of the four groups of samples did not change significantly.

The difference in hydrophobicity may be due to the hydrophobic nature of the Si–O bond and the fact that silicon blocks tend to enrich on the surface of epoxy resin during the curing process [24]. When the content of silicone exceeds 10 wt%, the silicone material on the surface tends to be saturated. Excessive silicone modifier may lead to worse compatibility, resulting in worse hydrophobicity.

### 3.5. FT-IR Spectra

**Figure 14:**
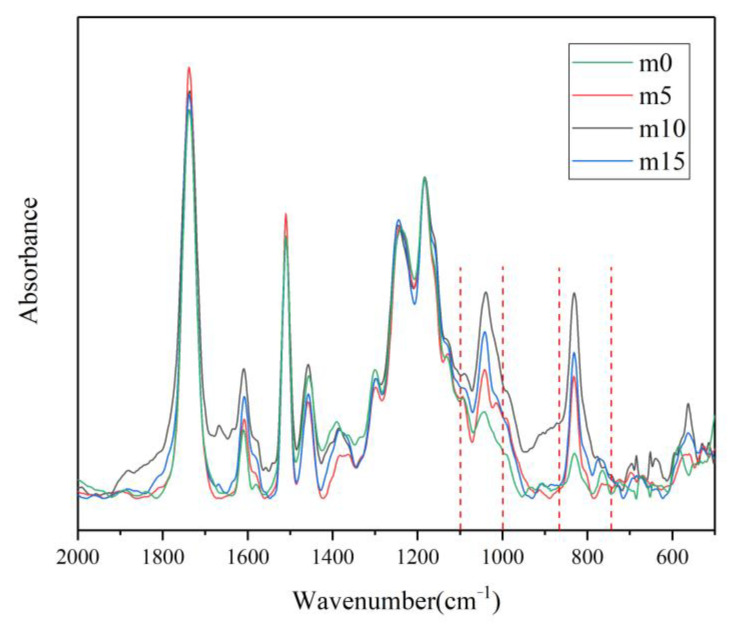
FT-IR spectra of the four groups of epoxy resin.

The FT-IR spectra of the four groups of epoxy resin are shown in Figure 14.

To minimize the error brought by the amount of sample used in the sample preparation process, the FT-IR spectra were normalized with ether bond. The 1100 cm^−1^–1000 cm^−1^ and 740 cm^−1^–870 cm^−1^ ranges contributed to the vibration of Si–O–Si and Si–C. There were dramatic increases in those two ranges after the modification, which confirm the grafting reaction. It is also noteworthy that while the Si–O–Si bond and Si–C bond increased with the content of the modifier under 10 wt%, they decreased after the content of the modifier exceeded 10 wt%. This may suggest that when the content exceeds 10 wt%, part of the modifier neither grafts on the main chain of the epoxy resin nor takes part in polymerization; instead, some modifier molecules exist in the system as impurities, resulting in the decrease in the anti-aging properties.

**Figure 15:**
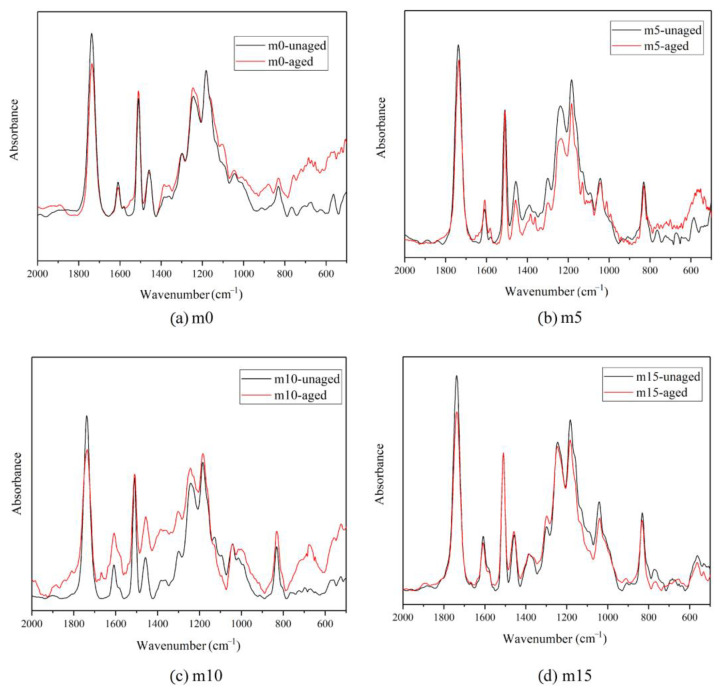
Change of FT-IR spectra after aging: (**a**) m0; (**b**) m5; (**c**) m10; (**d**) m15.

Figure 15 shows the change of the FT-IR spectra after the aging test. These spectra are modified with the phenyl group since it is the most stable group within the system and unlikely to change during the aging test. As shown in Figure 15, in all four groups, it can be observed that the peak at around 1740 cm^−1^ which is attributed to the carbonyl group dwindled during the aging process. This is because the carbonyl group is the most susceptible to hydrolysis. However, there were no obvious changes in the other peaks, indicating that the backbone of epoxy resin molecules is relatively stable. The changes in the peak around 1740 cm^−1^ by finding the ratio of the peak height before and after the hygrothermal aging are compared using Equation (4):

(4) $$\Delta H=\frac{H_{unaged}}{H_{aged}}$$

The results are summarized in Table 3. For all four groups, the ratios are almost the same, meaning the modifier did not change the hydrolysis rate of the carbonyl group. However, with the addition of the modifier, the content of the carbonyl group decreased, which could lead to better chemical stability.

### 3.6. FDS

**Figure 16:**
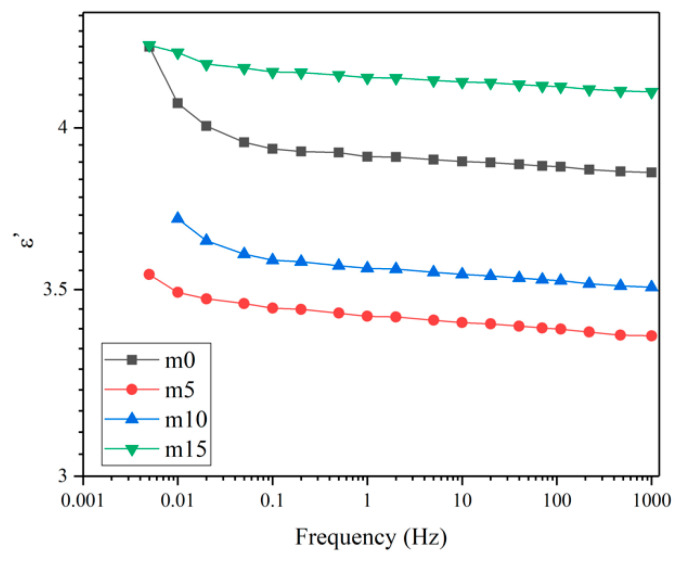
The real part of the complex dielectric constant of the four groups of unaged samples.

**Figure 17:**
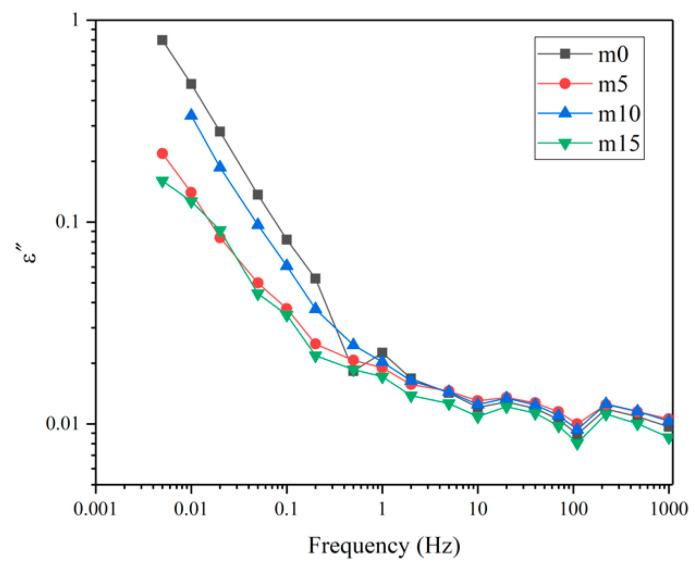
The imaginary part of the complex dielectric constant of the four groups of unaged samples.

**Figure 18:**
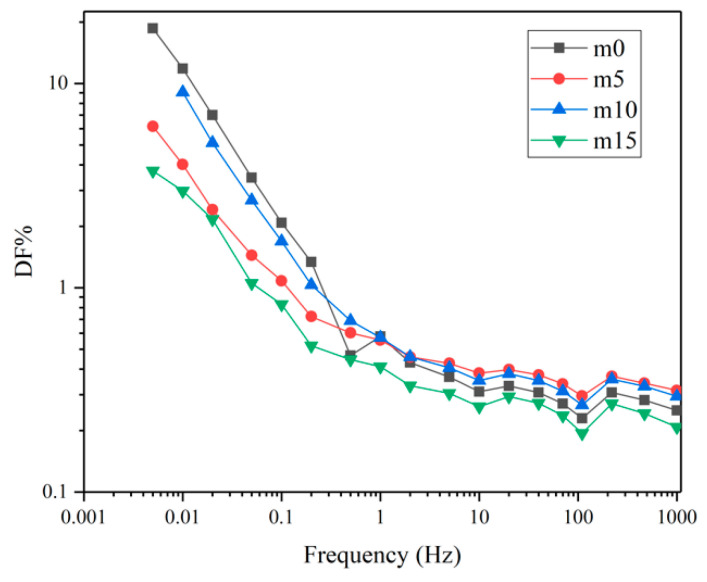
The dissipation factors of the four groups of unaged samples.

Unaged samples and samples aged for 32 days were selected to perform the FDS test. Figure 16 and Figure 17 show the variation of the real and imaginary part of the complex permittivity with the frequency of the four groups of unaged samples. Figure 18 shows the variation of the dissipation factor with the frequency of the four groups of unaged samples. The dissipation factor represents the ratio between active power and reactive power, and is defined by

(5) $$DF\%=\frac{\varepsilon^{\prime \prime }}{\varepsilon \prime }\times 100\%$$

The real part of the complex dielectric constant ε′ reflects the polarization of molecules. From Figure 16, it is obvious that ε′ decreases as the frequency increases, which is because the relaxation of molecules cannot keep up with the changes of the electric field. Comparing the curves of different groups of samples, at the same frequency, with the increase in modifier, the ε′ shows a decrease–increase trend. This is because the low polarity of diphenylsilanediol decreases the ε′ of the insulating material, while excessive diphenylsilanediol leads to worse compatibility, intensifying the polarization of the insulating material under the electric field.

Figure 17 and Figure 18 represent loss. At the same frequency, with the increase in modifier, the loss shows a decrease–increase–decrease trend. Under the electric field, the loss is mainly due to the polarization of dipoles and conductance. When the content of diphenylsilanediol is low, this modifier shows good compatibility with the epoxy resin. At 10 wt%, excessive diphenylsilanediol introduces more dipoles into the insulating material; thus, the loss increases. At 15 wt%, though dipoles continue to increase because of the fact that silicone materials have excellent insulation properties, the conductance of the insulating materials decrease; therefore, the material shows a lower ε″ and dissipation factor.

**Figure 19:**
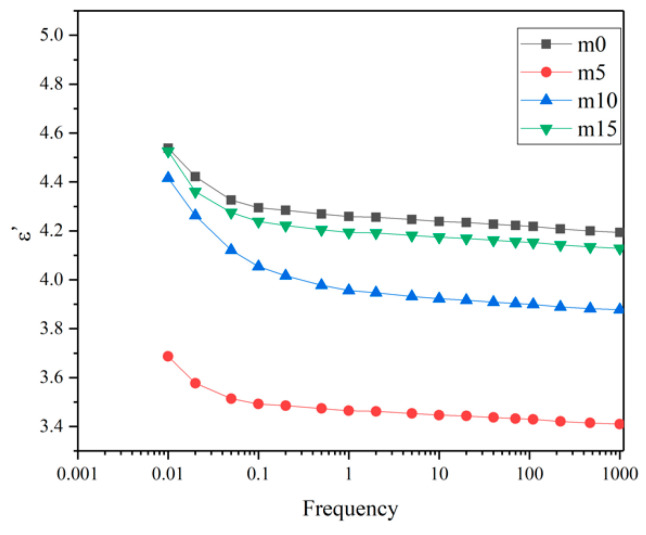
The real part of the complex dielectric constant of the four groups of aged samples.

**Figure 20:**
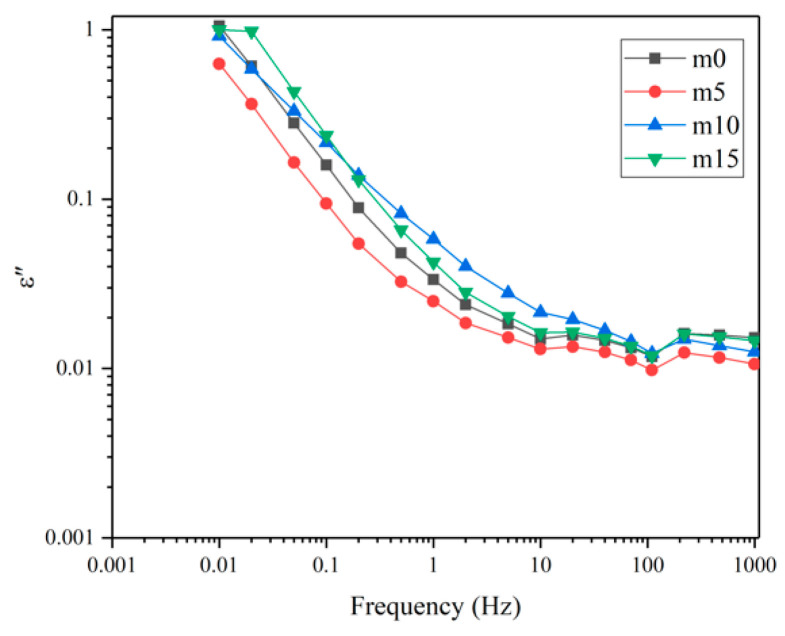
The imaginary part of the complex dielectric constant of the four groups of aged samples.

**Figure 21:**
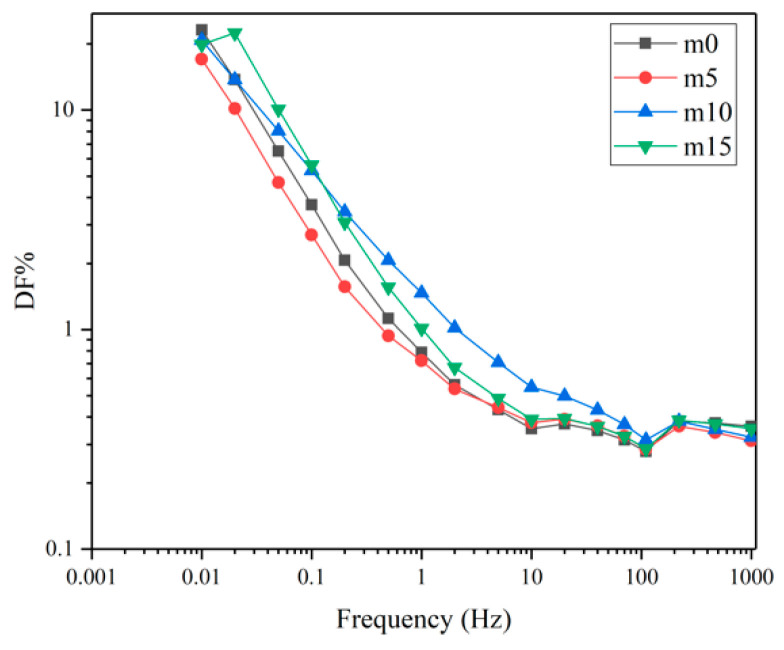
The dissipation factors of the four groups of aged samples.

Figure 19 and Figure 20 show the real and imaginary parts of the complex dielectric constant of the four groups of aged samples. Figure 21 shows the dissipation factor of the aged samples. During hygrothermal aging, hydrolysis may break the long chain and generate smaller molecules with stronger polarity; water molecules diffused into the system also have high polarity and relatively short relaxation time. Therefore, comparing Figure 16 and Figure 19, it can be observed that all curves moved upwards and became steeper under 0.1 Hz after hygrothermal aging. The comparison also reveals that during hygrothermal aging, the ε′ of m0 increased faster than other modified epoxy resin insulating materials; this may be due to the fact that although FT-IR analysis showed those materials hydrolyze at about the same rate, modified insulating materials contain less carbonyl groups, thus containing less molecules with high polarity after aging. The fact that m5 had the smallest increase in ε′ may be because it has the best compatibility, thus having fewer interfaces. Higher ε′ means worse distortion of the electric field in micropores within the system, and this may help to explain why the PDIV of m0 and m15 are obviously lower than those of m5 and m10.

Comparing Figure 17, Figure 18, Figure 20 and Figure 21, after aging, the loss increased. This is because the water molecules that diffused into the material became carriers and increased the conductance of the insulating material. It can also be noticed that after aging, four curves in Figure 20 and Figure 21 almost overlap, which indicates that although diphenylsilanediol could decrease the conductance of unaged samples, it has a limited effect on the conductance during the hygrothermal aging process.

## 4. Conclusions

In this paper, water absorption, PDIV and the surface contact angle of four groups of epoxy resins with different diphenylsilanediol contents under 32 days of hydrothermal aging conditions were studied to investigate the change of properties of silicone-modified epoxy resin. FT-IR, SEM and FDS were conducted to reveal the mechanism. The results are as follows:

1. Diphenylsilanediol can slow down diffusion speed and the generation and development of cracks and pores in epoxy resin samples under hygrothermal conditions to enhance insulating materials’ ability to withstand hygrothermal aging.
2. Hygrothermal aging has a great influence on PDIV. The decrease in PDIV in a hygrothermal environment is mainly influenced by the change of electrical properties of the material during aging and the size of cracks and pores in the material.
3. The introduction of diphenylsilanediol does not change the hydrolysis rate of the insulating material; the backbone of the silicone-modified insulating material remained stable during this experiment.
4. Diphenylsilanediol could reduce the number of molecules with high polarity in the system during the hygrothermal process but has a limited effect on the conductance of the insulating materials when aged.

This article found that PD behavior could be greatly influenced by hygrothermal aging. For future research, it might be interesting to extract more data from PD behavior, for example, PD intensity, and relate new PD data to hygrothermal aging. Moreover, finding the relation between the changes of mechanical properties and changes of dielectric properties may also bring interesting results.

## Figures and Tables

**Table 1:** EEW of the modified epoxy resin.

	m0	m5	m10	m15
**EEW (eq/100 g)**	52.013	51.273	51.985	52.028

**Table 2:** Fitting results of the four groups of samples.

	S	$m_{\infty }$	R^2^
m0	0.1232	1.078	0.98601
m5	0.0832	0.8112	0.96967
m10	0.0786	0.6077	0.97562
m15	0.1042	0.6532	0.98396

**Table 3:** Changes in carbonyl peak.

	m0	m5	m10	m15
**ΔH**	1.24	1.08759	1.21988	1.20769

## Data Availability

The data presented in this study are available on request from the first authors and corresponding author.
